# Melanoma Recurrence Prediction Using a Tissue-Free Epigenomic Molecular Residual Disease Assay: A Multicenter Prospective Observational Study (COSMOS-MEL01)

**DOI:** 10.1158/2767-9764.CRC-26-0388

**Published:** 2026-10-15

**Authors:** Kenjiro Namikawa, Dai Ogata, Eiji Nakano, Naoya Yamazaki, Azusa Komada, Akira Takahashi, Shusuke Yoshikawa, Chiemi Notake, Ryuta Mitani, Takayuki Yoshino, Yoshiaki Nakamura

**Affiliations:** 1 Department of Dermatologic Oncology, National Cancer Center Hospital, Tokyo, Japan.; 2 Clinical Research Support Office, National Cancer Center Hospital, Tokyo, Japan.; 3 Department of Dermatologic Oncology, National Cancer Center Hospital East, Kashiwa, Japan.; 4 Department of Dermatology, Shizuoka Cancer Center, Shizuoka, Japan.; 5 Translational Research Support Office, National Cancer Center Hospital East, Kashiwa, Japan.; 6 Department of Gastroenterology and Gastrointestinal Oncology, National Cancer Center Hospital East, Kashiwa, Japan.

## Abstract

Despite recent adoption of perioperative therapy for high-risk melanoma, its overall survival (OS) benefit remains unclear, highlighting the need for biomarkers to identify patients likely to benefit from treatment. Detection of circulating tumor DNA (ctDNA) after definitive treatment predicts recurrence, and emerging molecular residual disease (MRD) assays may influence management. We validated a tissue-free MRD assay in 48 patients with clinical stage II to III melanoma treated with surgery (52% nonacral cutaneous, 31% acral, and 17% mucosal). The assay detects MRD through bioinformatic analysis of methylation signals across >20,000 epigenomic regions. Plasma samples (*n* = 281; 280 successfully reported) were prospectively collected before surgery, at day 28, and every 3 to 6 months thereafter. Associations among MRD detection, recurrence, and OS were analyzed. At a median follow-up of 34.6 months with 27 recurrence events, postoperative surveillance sensitivity for distant recurrence was 81% with 100% posttreatment specificity. The median lead time between MRD detection and radiographic recurrence was 70.5 days. Surveillance MRD detection was associated with increased recurrence risk [hazard ratio (HR), 26.55; 95% confidence interval (CI), 2.86–246.58; *P* = 0.004]. Similarly, MRD detection 28 days postoperatively independently predicted recurrence (HR, 11.70; 95% CI, 3.09–43.76; *P* < 0.001) and death (HR, 31.14; 95% CI, 5.37–221.1; *P* < 0.001). Tissue-free epigenomic MRD detection was sensitive, specific, and significantly associated with recurrence and melanoma-related death. These findings support the use of this assay in melanoma as a rapid, noninvasive approach for earlier identification of patients at high risk for recurrence, enabling refined treatment and surveillance strategies.

**Significance:**

Prognostic tools for high-risk melanoma rely on baseline factors with limited dynamic risk assessment. A tissue-free MRD assay demonstrated high sensitivity and specificity for distant recurrence detection, identifying molecular relapse before radiographic recurrence. Postoperative MRD detection was strongly associated with recurrence and melanoma-related death, enabling refined treatment and surveillance strategies.

## Introduction

Cutaneous melanoma is a substantial health burden, with an estimated 332,000 new cases and nearly 59,000 deaths worldwide in 2022 (1). Despite advances in surgical techniques and the use of adjuvant or neoadjuvant immune checkpoint inhibitors (ICI) and targeted therapies, recurrence risk remains substantial in resected stage II to III disease (2, 3). At the same time, a definitive overall survival (OS) benefit of these perioperative therapies has not yet been established, and patients cured by surgery alone may receive unnecessary systemic therapy, exposing them to treatment-related toxicities and raising healthcare costs without clinical benefit. Current paradigms to assess recurrence risk and systemic treatment benefit are insufficient, and accurate blood-based biomarkers to assess for the presence of residual disease or to monitor for recurrence are lacking (4–7).

Postoperative surveillance includes clinical examination and intermittent radiographic imaging (CT, PET/CT, MRI, and/or ultrasound) that has limited sensitivity, particularly for early asymptomatic relapse, and exposes patients to repeated radiation and the risk for incidental/nonspecific findings that can result in unnecessary anxiety and additional workup (8–11). Guidelines and individual clinical practice vary owing to the unpredictable recurrence patterns and a lack of high-quality data to define optimal imaging strategies. Monitoring tools capable of dynamically stratifying recurrence risk over time are needed.

Circulating tumor DNA (ctDNA) has emerged as a promising approach for detecting molecular residual disease (MRD) and monitoring disease dynamics (12). Multiple studies demonstrate that ctDNA detection can precede clinical or radiographic relapse, often by several months (13–19). Existing studies have predominantly focused on advanced disease or assays that track a limited number of biomarkers or require tumor tissue sequencing, which can delay testing or exclude patients if tissue is unavailable. Tissue-free (plasma-only) ctDNA assays have emerged as a sensitive and specific predictor of recurrence, overcome these logistical barriers, and offer a scalable strategy for MRD detection and posttreatment monitoring (20–24). In this study, we evaluated the clinical performance of a tissue-free epigenomic MRD assay for surveillance in patients with resected stage II to III melanoma.

## Materials and Methods

### Study design

COnquer Solid Malignancies by blOod Screening MELanoma-01 [COSMOS-MEL01; registered at the UMIN Clinical Trials Registry (UMIN000042040)] is an ongoing multicenter prospective observational study in Japan. Eligible participants were ≥20 years of age with clinical stage II to III melanoma (cutaneous or mucosal) diagnosed within 60 days of enrollment, who were treatment naïve with planned surgical resection and had no radiographic evidence of distant metastasis.

From July 2021 through March 2023, 53 patients were enrolled. This interim analysis included samples available through December 31, 2024 (clinical data lock June 30, 2025). Clinical data were obtained from medical records. Recurrence status was determined by standard diagnostic imaging and procedures. The study was conducted in accordance with the Declaration of Helsinki and the Japanese Ethical Guidelines for Medical and Biological Research Involving Human Subjects. Approval was obtained from the Institutional Review Board of the National Cancer Center Japan (approval number 2020-408), and all patients provided written informed consent.

Blood was collected before surgery, ∼28 days postoperatively, and every 3 to 6 months for up to 3 years or until disease recurrence, with additional samples obtained at recurrence when feasible (Supplementary Fig. S1). Imaging was performed approximately every 6 months in accordance with Japanese Dermatological Association Guidelines. Plasma was stored and analyzed retrospectively, and results were not made available to treating physicians. Test results were generated blinded to clinical outcomes data. This interim analysis was designed to evaluate the postoperative samples to align with validation of the assay’s intended use, that is, postoperative detection of ctDNA to inform patient risk of recurrence. The prognostic value of preoperative samples will be reported in a separate study.

### ctDNA assay

MRD was analyzed using the tissue-free Guardant Reveal assay (Guardant Health) which evaluates >20,000 cancer-associated differentially methylated regions (DMR) and control regions. A bioinformatic algorithm trained to detect melanoma-specific DMRs distinguishes tumor-derived signals from background cell-free DNA (cfDNA) to classify samples as ctDNA positive or negative according to a predefined statistical threshold. The melanoma-specific algorithm was trained from an independent set of >500 samples from individuals with melanoma and >6,000 samples from individuals without cancer. Tumor fraction is calculated by normalizing cancer-specific DMRs with matched control regions to estimate the proportion of tumor-derived cfDNA molecules in each sample.

### Statistical analysis

The primary objective was to determine the clinical performance for postoperative surveillance, including assessment of sensitivity, specificity, and lead time for detecting distant metastatic recurrence in patients with stage II to III melanoma. The surveillance population was defined as having at least two postoperative timepoints, although patients with early relapse within 6 months of surgery were included if only one sample was available given opportunity to collect a second sample was limited.

The Kaplan–Meier method was used to estimate distant recurrence-free interval (d-RFI), recurrence-free interval (RFI), and OS stratified by ctDNA detection during surveillance (ever vs. never detected). To account for potential differences in proportional hazards over time and to avoid immortal time bias, we conducted landmark survival analyses at 12, 18, and 24 months postoperatively. This approach ensured that ctDNA classification was based on information available by that timepoint by conditioning on patients who remained event free at each landmark. Survival differences were assessed using log-rank tests and analytic methods robust to sample size, multiple comparisons, and the influence of confounders: the Holm multiplicity adjustment, Kaplan–Meier survival probability differences with both Greenwood-based and bootstrap 95% confidence intervals (CI), and adjusted Cox proportional hazards models that included age category (<65 vs. ≥65), diagnosis type (cutaneous or mucosal), stage, sex and use of adjuvant therapy as covariates.

We also assessed d-RFI, RFI, and OS differences based on ctDNA status at the single timepoint collected at day 28 postoperatively, adjusting for age, stage, diagnosis, sex, and use of adjuvant therapy.

### Study population

Of 53 enrolled patients, five were excluded from both postoperative analyses: one withdrew, one had stage IV disease at initial diagnosis, one had no postresection plasma samples available, and two had R2 resections. Of the remaining 48 patients, 47 were evaluable for the surveillance analysis (1 was excluded because of having only a single postoperative timepoint), 47 were evaluable for the day 28 analysis (1 was excluded because of the day 28 sample being collected after adjuvant treatment start), and 46 were included in both analyses (Supplementary Fig. S2). Samples with no clinical follow-up after collection were excluded (*n* = 1). Of note, both R2 resected patients had ctDNA detected at all available postoperative timepoints, and both died from melanoma.

Demographic and clinical characteristics are detailed in Supplementary Table S1. All received up-front surgery, and none had neoadjuvant therapy. The cohort included 8 patients with mucosal melanoma, 15 with acral lentiginous melanoma, and the remainder had nonacral cutaneous melanoma. More than half of patients (55%) had pathologic stage IIIB/C disease. One patient with clinical stage III/pathologic stage IB had a single postoperative timepoint and was included in the day 28 analysis. Approximately 19% had a *BRAF* V600E/K mutation. A total of 28 (58%) patients received adjuvant therapy (3 received dabrafenib + trametinib, and the remaining 25 received treatment with anti–PD-1 antibodies). At the time of data cutoff (median follow-up of 34.6 months), 27 patients were diagnosed with recurrent disease (16 distant, 10 regional, and 1 local). There were 11 deaths, all related to recurrent melanoma. All nonrecurred patients had at least 26 months of follow-up.

## Results

### Surveillance analysis

**Figure 1.**
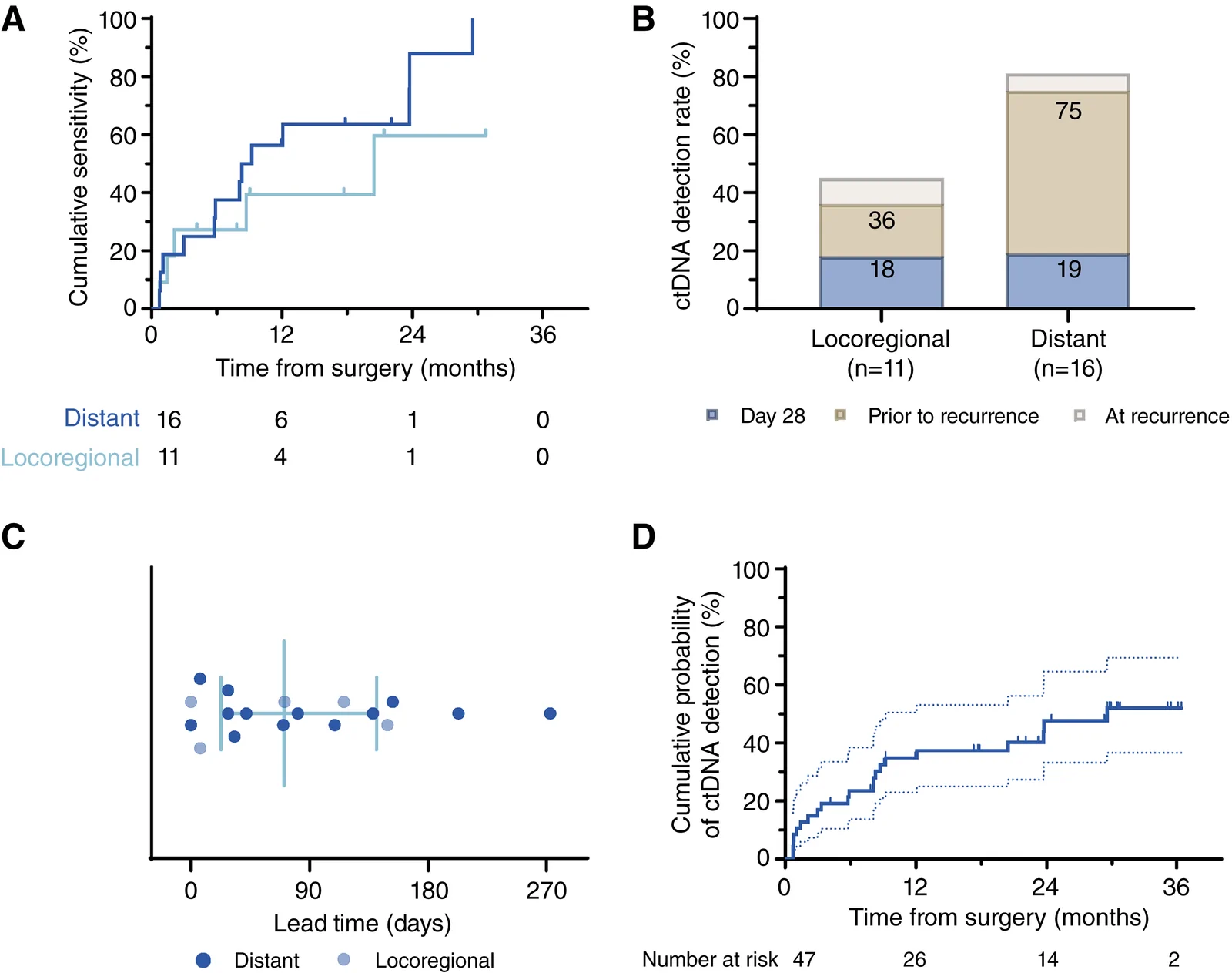
ctDNA detection increases with surveillance sampling and is associated with lead time prior to recurrence. **A,** Kaplan–Meier estimate of cumulative sensitivity of ctDNA detection with serial sampling in patients with recurrence. **B,** Timing of first ctDNA detection (day 28 postoperatively, after day 28 but prior to recurrence, or at recurrence) among 27 patients with recurrence based on anatomic location of recurrence. **C,** The median lead time among 18 samples with ctDNA detected in patients with recurrence was 70.5 days (IQR, 22.8–140.8 days) but ranged up to 273 days. **D,** Kaplan–Meier estimate of cumulative probability of ctDNA detection in all patients with stage II–III melanoma.

**Figure 2.**
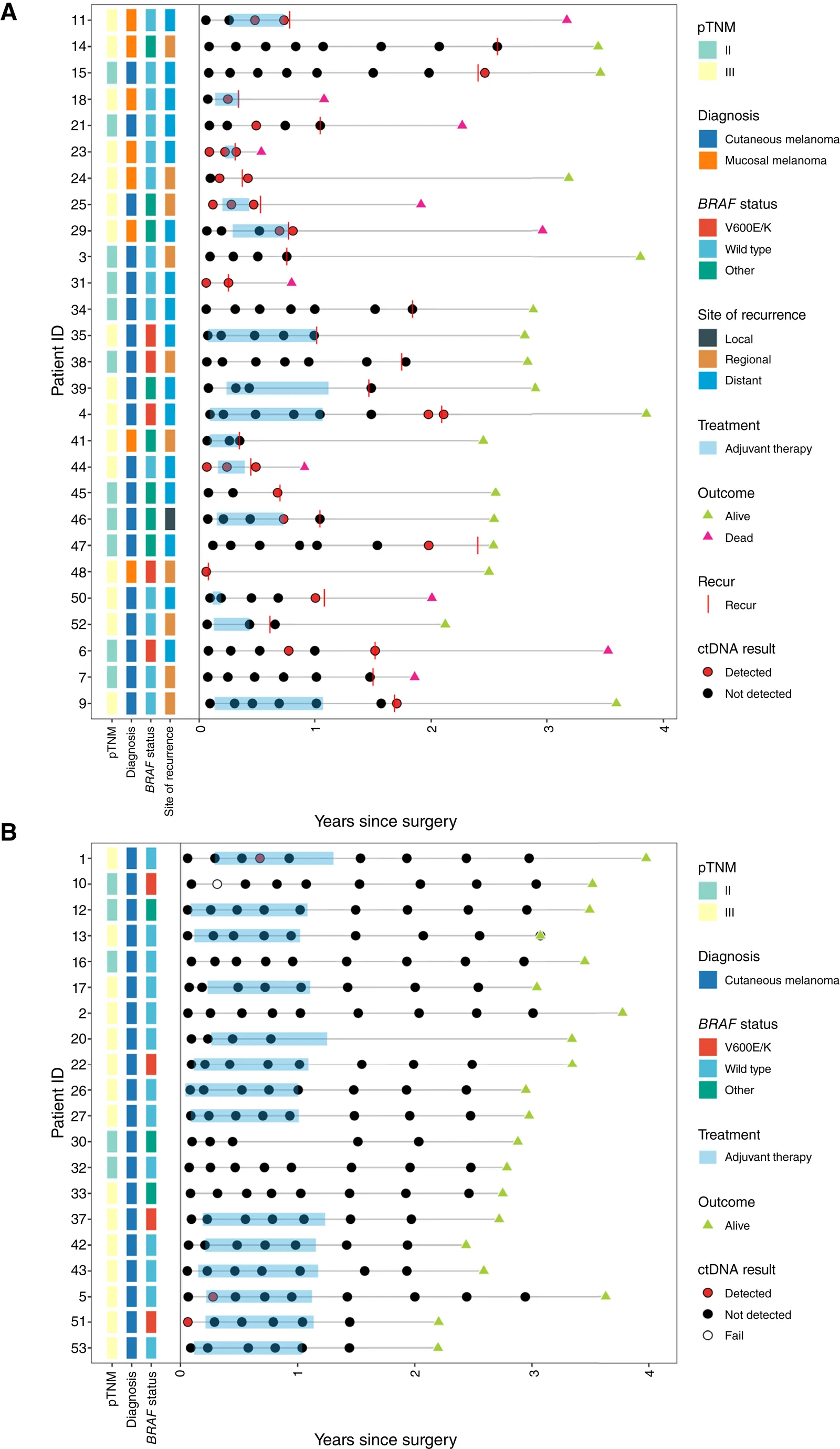
Swimmer plots depicting ctDNA results for patients included in the surveillance analysis. **A,** Patients with recurrence; (**B**) patients with no recurrence at data cutoff. pTNM, pathologic tumor–node–metastasis.

Of 47 patients evaluable for the surveillance analysis, 278 of 279 (99.6%) available samples were successfully analyzed (Supplementary Fig. S1). The median sampling per patient was 6 (range, 1–9). Cumulative sensitivity for ctDNA across postoperative timepoints was 81% (13/16) in patients with distant recurrence and 45% (5/11) in patients with locoregional recurrence (Fig. 1A). Sensitivity remained at 81% (13/16) and 45% (5/11) for distant and locoregional recurrence, respectively, among the subset of posttreatment samples (i.e., excluding samples collected prior to the end of adjuvant therapy for those who received it). ctDNA was detected prior to clinical detection of recurrence in 75% of patients with distant and 36% of patients with locoregional recurrence, whereas two patients had detectable ctDNA at the same time as the clinical recurrence diagnosis (Fig. 1B). ctDNA detection preceded clinical relapse detection by a median of 70.5 days [interquartile range (IQR), 22.8–140.8 days; range up to 273 days] and was similar for locoregional or distant relapse (Fig. 1C). ctDNA was detected in at least one postoperative timepoint in 45% (21/47) of patients, increasing steadily during surveillance from 13% at 1 month after surgery to more than 40% within 2 years (Fig. 1D). Patient-level longitudinal ctDNA results are shown for patients with recurrence in Fig. 2A and for patients without recurrence at data cutoff in Fig. 2B. Among the 19 patients without recurrence, specificity was 100% across 84 samples collected after completion of all treatment (surgery or adjuvant therapy if given). Rates of ctDNA detection, sensitivity, and posttreatment specificity by stage and for patients with mucosal melanoma are provided in Supplementary Table S3.

**Figure 3.**
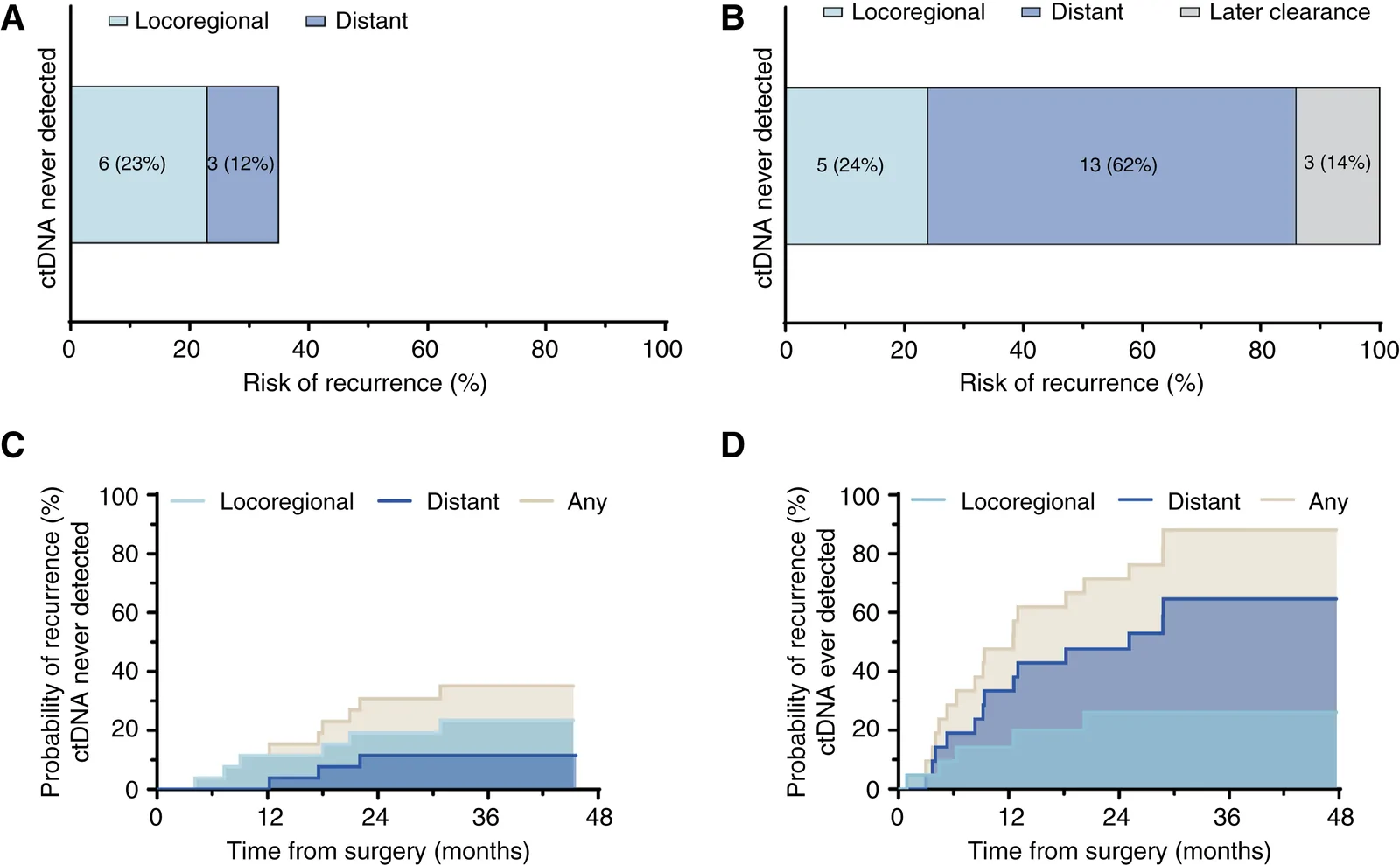
Risk for recurrence based on ctDNA detection during surveillance. Overall observed rate of relapse at data cutoff (**A** and **B**) and Kaplan–Meier estimates of probability of recurrence over time (**C** and **D**) by anatomic site based on longitudinal ctDNA detection after surgery. **A** and **C,** Never detected; (**B** and **D**) ever detected. Three patients with detectable ctDNA before or during adjuvant therapy cleared ctDNA at later timepoints without recurrence.

We next compared recurrence patterns by longitudinal ctDNA detection status. Of the 26 patients who remained negative with surveillance sampling, 3 (12%) developed distant recurrence and 6 (23%) developed locoregional recurrence (Fig. 3A). In contrast, of 21 patients with detectable ctDNA at any timepoint postoperatively, 18 (86%) experienced recurrence, 13 of which were distant recurrences (Fig. 3B). Three patients with detectable ctDNA did not recur, including a patient whose day 28 postoperative sample was positive who subsequently received 11 months of adjuvant nivolumab and had five subsequent surveillance samples with undetectable ctDNA and two patients whose only positive sample occurred during adjuvant immunotherapy and then subsequently cleared. These three cases are consistent with clearance of MRD with adjuvant therapy. In total, six of nine patients with detectable ctDNA before or during adjuvant therapy recurred, whereas all 17 patients with ctDNA detected after treatment recurred. Recurrence over time according to longitudinal ctDNA detection status is shown in Fig. 3C and D.

**Figure 4.**
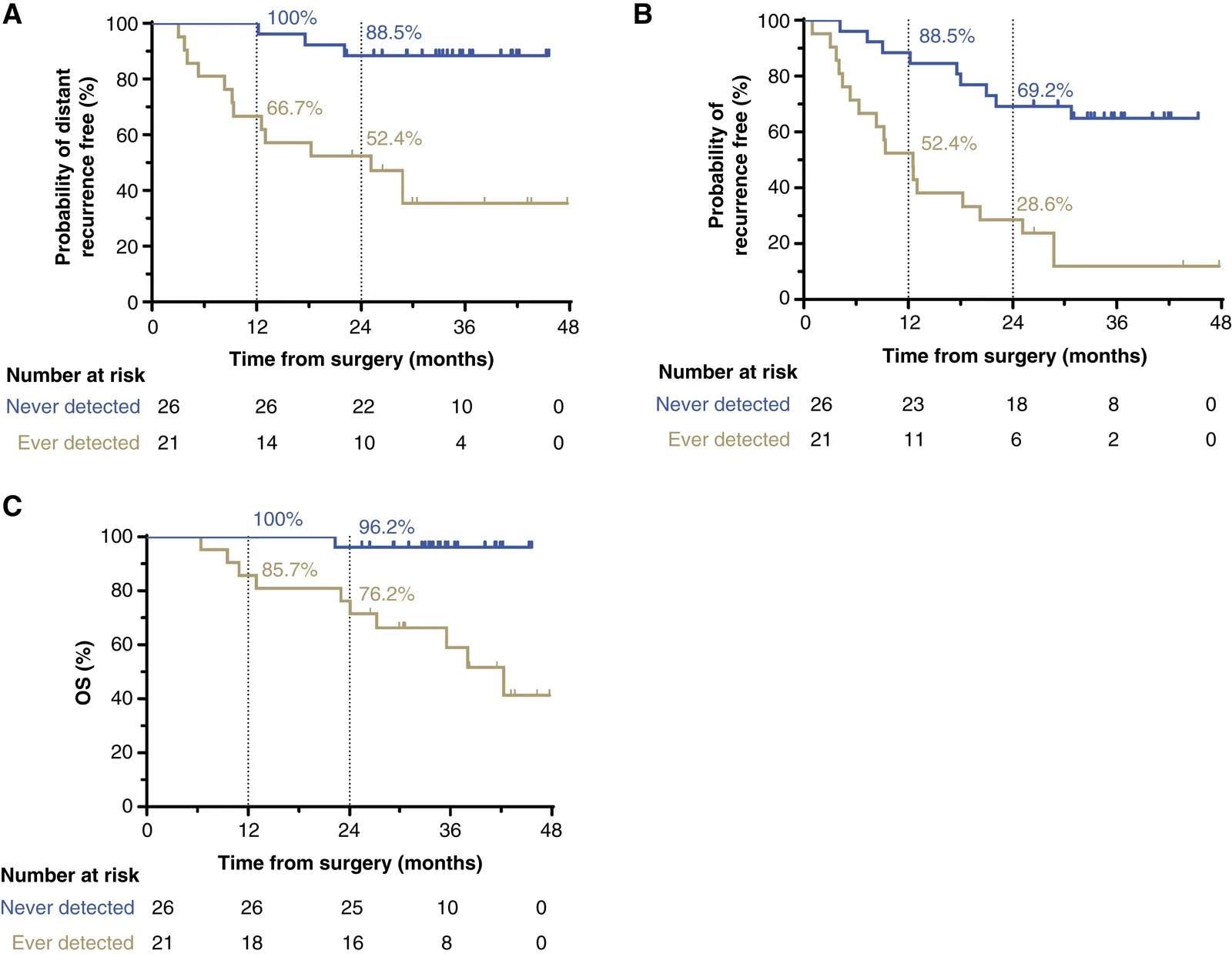
Detectable ctDNA during surveillance is associated with higher risk for recurrence and death from melanoma. Kaplan–Meier estimates of probability of distant recurrence (**A**), any recurrence (**B**), or OS (**C**) based on whether ctDNA was ever or never detected during longitudinal surveillance after surgery. HRs and *P* values corresponding to the landmark survival analysis at 12, 18, and 24 months after surgery can be found in Supplementary Table S4.

Statistically, detection of ctDNA at any timepoint during surveillance was associated with significantly worse outcomes after controlling for clinicopathologic variables (Fig. 4A–C; Supplementary Table S4). Patients with ctDNA detected had substantially lower d-RFI compared with those without ctDNA detection, with absolute differences ranging from 33.3% to 36.1% between 12 and 24 months from surgery using significant log-rank tests (*P* = 0.006–0.026). Although the Holm-adjusted *P* value did not reach statistical significance for each of the three d-RFI landmark survival analyses, the adjusted Cox model at 12 months demonstrated an increased risk of distant recurrence associated with ctDNA detection [adjusted hazard ratio (HR), 8.66; 95% CI, 1.74–43], with similar results observed using bootstrap estimation. Adjusted HR estimates at these later landmarks were unstable because of limited number of events. For RFI, absolute differences ranged from 36.1% to 40.7% across 12, 18, and 24 months of follow-up (Holm-adjusted *P* ≤ 0.037). The adjusted Cox model at 12 months confirmed a significantly increased risk of recurrence (adjusted HR, 5.16; 95% CI, 1.53–17.36), which was robust to bootstrap resampling, with an even higher magnitude of the association observed at 18 months that was significant despite having relatively few events (adjusted HR, 26.55; 95% CI, 2.86–246.58). OS was consistently lower among patients with ctDNA detected during surveillance. Absolute OS differences ranged from 14.3% to 20% across 12, 18, and 24 months, with significant log-rank tests (*P* = 0.015–0.031).

As expected, samples collected from patients with recurrence had a significantly higher number of detected DMRs than those without recurrence (Wilcoxon rank-sum test, *P* = 1.64 × 10^−3^; Supplementary Fig. S3). Additionally, all samples that passed the threshold for reporting as ctDNA detected were only observed in patients with recurrence or in samples collected prior to completion of definitive therapy. The median number of DMRs detected per positive sample in this study was 1,108 (IQR, 873–1,342). The median tumor fraction observed among 33 ctDNA-positive samples was 0.003 (0.3%; range, 0.006%–41.7%). In patients with multiple positive postoperative samples (*n* = 9), ctDNA quantity generally increased prior to recurrence (Supplementary Fig. S4).

### Prognostic value of the day 28 timepoint

**Figure 5.**
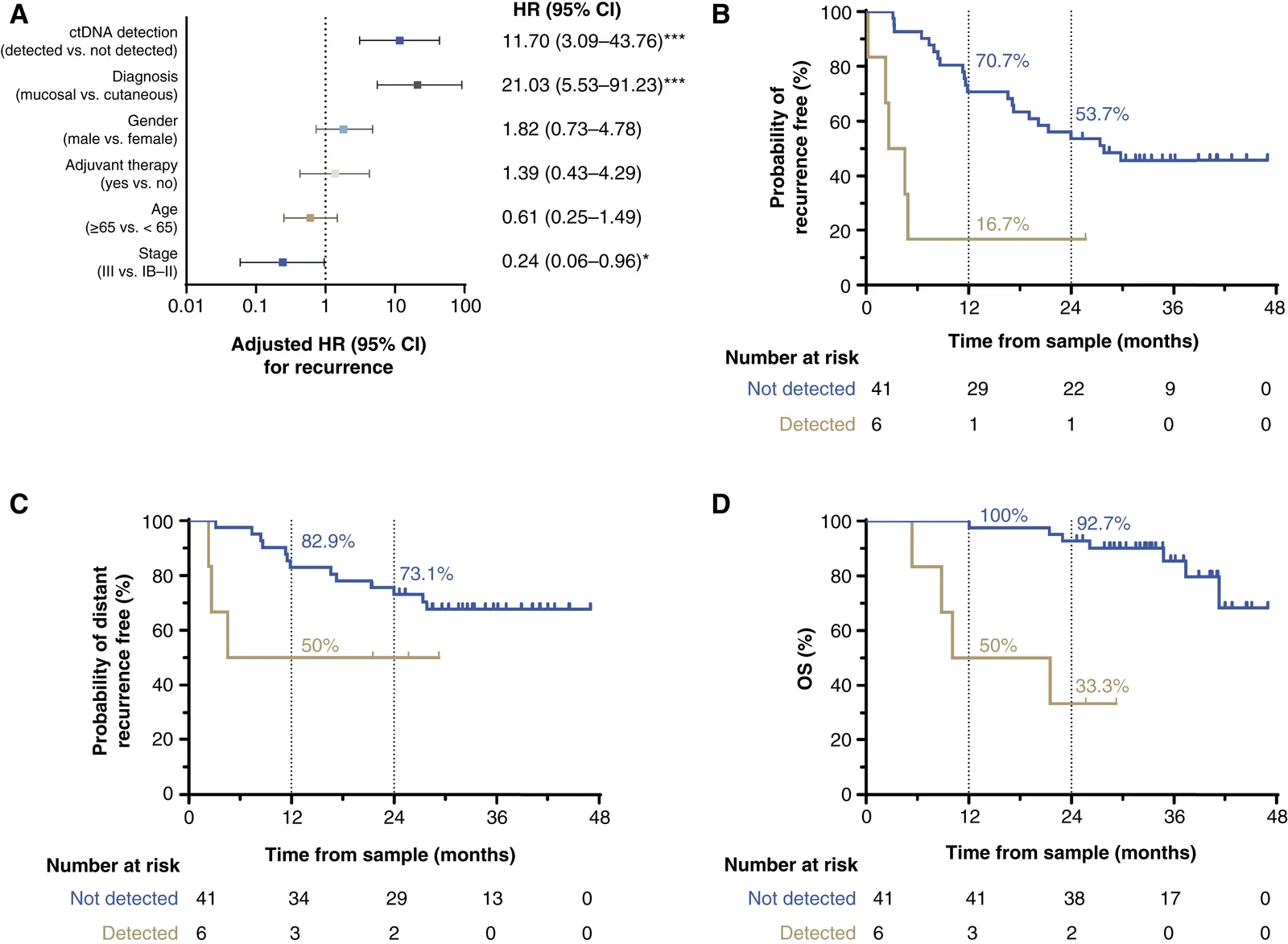
Detectable ctDNA at day 28 after surgery is associated with higher risk for recurrence and death from melanoma. **A,** Cox proportional hazard analysis of clinical factors associated with recurrence (***, *P* < 0.0001; *, *P* < 0.05) and Kaplan–Meier estimates for (**B**) any recurrence, (**C**) distant recurrence, and (**D**) OS based on ctDNA detection 28 days after surgery. HRs and *P* values can be found in Supplementary Table S5.

The day 28 cohort included 47 patients who had a sample collected ∼4 weeks after surgery, prior to adjuvant treatment start, and who had at least one follow-up visit. The median time from surgery to sample collection was 29 days (range, 21–43 days). A total of 6 (13%) patients had detectable ctDNA at this timepoint, which was strongly prognostic for recurrence (*P* < 0.001). Five of the six ctDNA-detected cases recurred, all within 6 months from surgery and despite the use of adjuvant treatment in three patients. The one ctDNA-positive patient who did not recur was subsequently treated with nivolumab, had later clearance of ctDNA, and remained disease free at 26 months as described above. The median RFI from the time of sample collection was 3.6 months [95% CI, 2.3–not evaluable (NE)] for day 28 ctDNA-positive patients versus 27.8 months (95% CI, 19.1–NE) in ctDNA-negative patients. After adjustment for stage, adjuvant therapy, age, sex, and diagnosis type, ctDNA detection remained a strong independent predictor of recurrence (adjusted HR, 11.70; 95% CI, 3.09–43.76; *P* < 0.001) along with mucosal versus cutaneous diagnosis type (Fig. 5A and B; Supplementary Table S5). All eight patients with mucosal melanoma recurred, with two having ctDNA detected at the day 28 timepoint and four having ctDNA detected during surveillance.

ctDNA detection at day 28 showed a trend toward worse d-RFI that did not reach statistical significance (adjusted HR, 3.87; 95% CI, 0.79–15.14; *P* = 0.089), although the analysis was limited by fewer events (*n* = 16; Fig. 5C; Supplementary Table S5). For patients with undetectable ctDNA at the day 28 timepoint, the likelihood of being recurrence free and distant recurrence free at 12 months from the time of sample collection was 70.7% (95% CI, 54.3%–82.2%) and 82.9% (95% CI, 67.5%–91.5%), respectively. For patients who continued to have undetectable ctDNA through the 12-month longitudinal sample, the likelihood of remaining recurrence free and distant recurrence free at 24 months was 80.8% (95% CI, 67%–97.4%) and 92.6% (95% CI, 83.2%–100%), respectively.

ctDNA detection at the day 28 timepoint was also strongly associated with inferior OS (log-rank *P* < 0.001; Fig. 5D; Supplementary Table S5). The median OS from the time of sample collection was 15.8 months (95% CI, 5.4–NE) for ctDNA-positive patients and was not reached in ctDNA-negative patients. Four of the ctDNA-positive patients (66.7%) died from melanoma within 2 years of surgery. In contrast, the OS rates for ctDNA-negative patients at 12 and 24-months following the day 28 sample collection were 100% and 92.7% (95% CI, 79%–97.6%), respectively. In multivariable analysis, ctDNA detection at day 28 was associated with a greater than 30-fold increased risk of death (HR, 31.14; 95% CI, 5.37–221.2; *P* < 0.001). For patients who continued to have undetectable ctDNA through the 12-month longitudinal sample, the likelihood of remaining alive at 24 months was 96.3% (95% CI, 89.4%–100%).

## Discussion

In this prospectively designed multicenter study, a tissue-free epigenomic ctDNA assay identified a substantial proportion of postoperative ctDNA-positive patients with stage II to III melanoma which was associated with high risk of recurrence and poor survival. ctDNA was often detectable months before radiographic evidence of relapse. These data highlight the value of longitudinal monitoring in this population given the significant increase in ctDNA detection over time.

Integrating this technology after resection could offer a personalized risk-informed approach to radiographic surveillance and could further personalize existing tools of conditional risk based on pooled data from large pivotal trials in stage III disease (25). A recent global consensus survey of the Melanoma World Society Study group identified detection of MRD and guiding relapse surveillance as highly valuable clinical applications of ctDNA in the treatment of melanoma and 82% of respondents were supportive of integrating ctDNA into routine posttreatment surveillance (26). Current surveillance strategies rely on static information from clinicopathologic staging which lacks precision with regard to recurrence risk over time. In contrast, ctDNA monitoring offers dynamic risk stratification and could increase the efficiency and effectiveness of radiographic imaging by identifying high-risk patients who may benefit from a more frequent cadence, potentially enabling earlier identification and intervention of lower burden disease that may be more amenable to curative-intent therapy (9). ctDNA demonstrated high sensitivity for distant relapses, reflecting higher shedding rates in systemic disease, whereas sensitivity for locoregional recurrence was more modest—consistent with observations from other studies of melanoma and other solid tumors that total disease burden affects ctDNA detection (27–29). Notably, persistently ctDNA-negative patients had low rates of distant recurrence with promising OS trends, which may offer reassurance for these patients. Taken together, these findings support integration of ctDNA as an adjunct to imaging, allowing risk-adapted tailoring of surveillance frequency and intensity. Furthermore, specificity was high, and transient positives before completion of adjuvant therapy likely represented clearance of true minimal residual disease. Our data and others suggest that systemic treatment may effectively clear some patients’ ctDNA and prevent or delay disease progression (19, 30, 31).

The prognostic value of an early ctDNA measurement at day 28 after surgery reinforces the potential of ctDNA for informing postoperative decisions. Three of the four ctDNA-positive patients at this timepoint experienced rapid recurrence despite adjuvant therapy, whereas both ctDNA-positive patients who did not receive adjuvant treatment recurred. Further two thirds of the day 28 ctDNA-positive patients died of melanoma. Our data suggest that ctDNA positivity at this early timepoint identifies a subset of patients with biologically aggressive residual disease who may require alternative management approaches. Such patients could be good candidates for clinical trials investigating new therapeutic strategies and may benefit from close radiographic surveillance, although standard treatment may effectively clear some patients’ ctDNA. Although sensitivity for any relapse at the day 28 timepoint was modest, the distant recurrence risk and survival outcomes in ctDNA-negative patients (70.7% D-RFI and 92.7% OS at 2 years) suggest that postoperative ctDNA-negative status may identify a subset of patients with more favorable biology, even if ctDNA is not able to detect all relapses at this timepoint. ctDNA status may provide meaningful personalized risk estimates beyond staging that could provide reassurance to lower-risk patients and inform adjuvant therapy risk–benefit discussions. Although there are no data to support the safety of routine adjuvant therapy de-escalation for patients who are ctDNA negative, the test may inform patient counseling and shared decision-making. Toxicities are common in BRAF-directed therapies, and although they are unlikely to be permanent, they can have a significant impact on the quality of life. Approximately 37% of patients undergoing immunotherapy develop immune-related adverse events of any grade, with 7% experiencing grade 3 or higher events (32). Although this is manageable in many patients, the nature of these toxicities matters as they can be permanent and life-threatening. Furthermore, despite improvement in recurrence-free and distant metastasis-free survival, it is not yet clear whether OS is improved when immunotherapies are given in the adjuvant setting versus waiting to initiate them at the time of metastatic progression (33). Thus, postoperative ctDNA status may be highly relevant for those with questionable benefit from adjuvant systemic treatment or who are at increased risk for treatment-related complications. The evaluation of potential side effects and treatment benefits is highly individual, and some patients wish to undergo therapy regardless of toxicity even with small benefit, whereas others may prefer less invasive approaches, particularly if they have existing autoimmune disease, a high comorbidity burden, and/or concerns about fertility. More data are urgently needed to define benefit of adjuvant therapy based on postoperative ctDNA status to inform these personalized approaches. Strategies that incorporate serial ctDNA testing into adjuvant decision-making could also be considered to reduce the false-negative rate and to examine the safety and efficacy of delaying administration of adjuvant therapy until molecular evidence of disease becomes apparent along with the ability to reduce toxicity and overtreatment in patients who remain negative during serial follow-up.

ctDNA monitoring might be particularly useful in *BRAF*-mutant disease, in which the optimal scheduling of BRAF/MEK inhibitors and ICIs remains uncertain. Because only nine patients with *BRAF*-mutant melanoma were included in the present study, we are currently conducting COSMOS-MEL02, which specifically focuses on *BRAF*-mutant melanoma (UMIN000051210). Whether ctDNA clearance or emergence can guide this decision or enable timely switching of therapy is an important area of ongoing research. The phase III SERENA-6 trial in metastatic breast cancer demonstrated that *ESR1* mutation monitoring and switching therapy at the time of molecular evidence of progression significantly reduced the risk of disease progression or death compared with switching therapy at the time of radiographic progression (34). In melanoma, longitudinal studies demonstrate ctDNA as a sensitive and reliable marker of monitoring treatment outcomes and the advanced and adjuvant settings in patients treated with ICIs or BRAF/MEK inhibitors (16, 17, 19, 30, 35–40). The CAcTUS pilot study (NCT03808441) in metastatic melanoma investigated whether dynamic changes in ctDNA can guide the transition from targeted to immune therapy and whether ctDNA-defined responses to BRAF inhibition improve the efficacy of subsequent immunotherapy. The study demonstrated the feasibility of longitudinal ctDNA tracking within actionable timeframes and suggested that ctDNA dynamics could capture very early responses to therapy and enable early switching of patients with poor prognosis disease from induction targeted therapy to immune therapy (41). The study further suggested that ctDNA monitoring could be an early predictor of immunotherapy failure that could trigger therapy switching, although the small sample size precludes definitive assessment of whether early switch strategies improve outcomes. Our data suggest that a substantial proportion of stage II to III patients have emergent ctDNA detection in the adjuvant setting, where these types of early switching strategies could be considered. An important area in need of further validation is the use of ctDNA status as a surrogate endpoint, which has the potential to provide an early indication of treatment efficacy, expediting both clinical evaluation and regulatory approval of emerging therapies.

Although several other studies in resected melanoma have established ctDNA as a strong prognostic biomarker, there is substantial heterogeneity in study design, sampling strategies, and statistical approaches with relatively few reporting prognostic data in the longitudinal setting. A recent meta-analysis evaluated the prognostic performance of ctDNA across more than 3,000 patients with resected nonuveal or mucosal melanoma across 21 studies (42). Most patients had stage III disease, and more than 90% were evaluated with tumor-informed assays. The pooled postoperative ctDNA detection rate within 12 weeks of surgery was 15%, similar to the observed rate in this study (13%). The pooled HR for recurrence based on ctDNA detection within 12 weeks of surgery was 3.42 (95% CI, 2.51–4.65) for any recurrence, with strong association for distant metastases (HR, 5.38) and OS (HR, 3.55). The pooled specificity among studies reporting longitudinal postoperative sampling was high (96.3%) with modest sensitivity of 45.2%, with limited distinction between distant and locoregional recurrence detection and highly variable sampling cadence. In one of the largest retrospective analyses of real-world data employing high-density longitudinal sampling (∼7 collections per patient) using a commercially available tissue-informed assay in resected stage I to III melanoma, sensitivity for distant recurrence was substantially higher than for locoregional relapse (∼85% and ∼29%, respectively) with ∼97% sample-level specificity, similar to our observed rates of 81%, 45%, and 100%, respectively (14). Our study is an important contribution to this body of work as we are the first to prospectively assess a tissue-free assay that tracks thousands of tumor-specific methylation changes with a standardized, protocol-driven sampling cadence. Cross-study comparisons are challenging given that survival analyses are affected by statistical methodologic choices and the baseline recurrence risk of the population being studied and longitudinal performance is affected by sampling density and the distribution of recurrence sites. However, the tissue-free assay evaluated in this study demonstrates comparable performance with first-generation tissue-informed ctDNA approaches. Our study demonstrates that tissue-free assays can enable sensitive detection of MRD and confirms findings from other studies that ctDNA has improved performance for monitoring systemic disease and is strongly predictive of OS with more limited detection for locoregional relapse. The tissue-free approach is clinically relevant as it eliminates dependence on tissue availability, enabling rapid assessment and broad applicability across melanoma subtypes, including cases in which tissue acquisition and sequencing can be challenging, particularly relevant with the increasing shift to neoadjuvant therapy application. The high analytic success rate (99.6%) and the ability to detect low-level methylation signals (∼50% of positive samples were detected below 0.3% tumor fraction) support the feasibility of routine clinical implementation. The newer generation of ultrasensitive ctDNA assays employing more extensive tumor sequencing and higher numbers of variant tracking may improve sensitivity; however, published data in the postoperative stage II to III setting are not yet available.

This interim analysis has certain limitations. The sample size is modest, particularly for ctDNA-positive patients at the day 28 postoperative timepoint; however, large, clinically, and statistically significant differences were observed owing to the sizeable prognostic effect of ctDNA detection. Larger studies may be helpful to further characterize the prognostic impact of ctDNA across histologic and molecular subtypes and adjuvant therapy classes. Additionally, the study did not mandate therapeutic interventions based on ctDNA status, and the clinical utility of acting on ctDNA status is an area of ongoing study. Notably, the DETECTION-2 (ISRCTN13695430) and Clear-Me (NCT06319196) trials are investigating whether new therapeutic strategies can improve outcomes and are utilizing ctDNA-guided management following surgery (43, 44).

As melanoma management continues to move toward more personalized approaches, ctDNA-based MRD assessment represents a promising step toward more precise and effective care. Our findings support the clinical utility of this tissue-free assay as a rapid method to detect MRD and identify patients at exceptionally high risk of recurrence and death from melanoma. This noninvasive tool offers insights into therapeutic decision-making, clinical trial innovation, and more personalized surveillance strategies, helping address several areas of unmet need in the management of resectable melanoma.

## Supplementary Material

Supplementary Figure 1 Supplementary Figure 1

Supplementary Figure 2 Supplementary Figure 2

Supplementary Figure 3 Supplementary Figure 3

Supplementary Figure 4 Supplementary Figure 4

Supplementary Table 1 Supplementary Table 1

Supplementary Table 2 Supplementary Table 2

Supplementary Table 3 Supplementary Table 3

Supplementary Table 4 Supplementary Table 4

Supplementary Table 5 Supplementary Table 5

## Data Availability

Patient-level clinical information and test results are provided in Supplementary Table S2. Raw sequencing data are not publicly accessible to comply with patient consent restrictions; however, additional information can be obtained from the corresponding author upon reasonable request.
